# Is There a Relationship Between Prenatal Dexamethasone and Postnatal Fructose Overexposure and Testicular Development, Function, and Oxidative Stress Parameters in Rats?

**DOI:** 10.3390/ijms252313112

**Published:** 2024-12-06

**Authors:** Nataša Ristić, Slavica Borković-Mitić, Milica Manojlović-Stojanoski, Nataša Nestorović, Branko Filipović, Branka Šošić-Jurjević, Svetlana Trifunović, Bojan Mitić, Jovana Čukuranović-Kokoris, Slađan Pavlović

**Affiliations:** 1 Institute for Biological Research “Siniša Stanković”—National Institute of the Republic of Serbia, University of Belgrade, Bulevar Despota Stefana 142, 11108 Belgrade, Serbia; borkos@ibiss.bg.ac.rs (S.B.-M.); manojlo@ibiss.bg.ac.rs (M.M.-S.); rnata@ibiss.bg.ac.rs (N.N.); brankof@ibiss.bg.ac.rs (B.F.); brankasj@ibiss.bg.ac.rs (B.Š.-J.); lanat@ibiss.bg.ac.rs (S.T.); sladjan@ibiss.bg.ac.rs (S.P.); 2 Institute of Zoology, University of Belgrade—Faculty of Biology, Studentski Trg 16, 11158 Belgrade, Serbia; bojan@bio.bg.ac.rs; 3 Department of Anatomy, Faculty of Medicine, University of Niš, Bulevar Dr Zorana Đinđića 81, 18000 Niš, Serbia; jovana.cukuranovic.kokoris@medfak.ni.ac.rs

**Keywords:** dexamethasone, fructose, testicles, rats, oxidative stress parameters, prenatal, offspring

## Abstract

Prenatal glucocorticoid overexposure alters the developmental program of fetal reproductive organs and results in numerous changes that can lead to various disorders later in life. Moderate fructose consumption during childhood and adolescence may impair the development and function of reproductive organs. The aim of this study was to investigate the effects of prenatal dexamethasone (Dx) exposure in combination with postnatal fructose overconsumption on testicular development and function in fetal and adult male rat offspring. Pregnant female rats were treated with a subcutaneous injection of Dx at a dose of 0.5 mg/kg/day on gestation days 16, 17, and 18, and the effects on fetal growth and testicular development were analyzed. Spontaneously born male offspring were fed 10% fructose in drinking water until the age of 3 months. Prenatal exposure to Dx led to a reduction in fetal weight and testicular volume. However, testicular development normalized by adulthood, with testosterone levels decreasing. After moderate fructose consumption, impaired redox homeostasis and structural changes in the testicles and decreased testosterone levels were observed, indicating reduced testicular function. The results suggest that the synergistic effect of prenatal Dx exposure and moderate postnatal fructose consumption leads to more deleterious changes in testicular tissue.

## 1. Introduction

Changes in the intrauterine environment caused by maternal stress, malnutrition, or abnormal hormone levels may alter the developmental program of fetal organs and induce a set of permanent fetal adaptive changes. These changes lead to an altered phenotype in adulthood and a predisposition to various disorders, syndromes, and diseases in later life [1]. Such observations are studied within the concept of developmental programming [2].

Glucocorticoids modulate cell proliferation and differentiation in the tissues during fetal development and are a crucial factor in the maturation of the organism and preparation for birth [3]. During most of the pregnancy, the glucocorticoid level in the fetal circulation is maintained within the physiological range by the activity of 11β-hydroxysteroid dehydrogenase type 2 [4]. However, maternal stress or treatment with synthetic glucocorticoids can lead to fetal overexposure to glucocorticoids. Systemic overexposure to glucocorticoids induces fetal adaptive mechanisms that might be disadvantageous throughout the life cycle [5].

Dexamethasone (Dx), a synthetic glucocorticoid, is commonly used during pregnancy to treat certain maternal conditions or a persistent risk of preterm birth, to promote fetal tissue differentiation and maturation, and to prepare the fetus for postpartum survival [6,7]. Because Dx crosses the placental barrier [8], its use results in fetal overexposure to glucocorticoids, with phenotypic outcomes associated with low birth weight [9]. In addition, the National Institutes of Health (Bethesda, MD, USA) recently recommended using Dx in the treatment of pregnant patients, with COVID-19 significantly increasing the exposure of pregnant women to Dx [10]. Therefore, there was a need to develop animal models that would allow us to understand the current and long-term consequences of overexposure to glucocorticoids during fetal development and for adult health and the quality of life. The field of reproductive biology is fascinating in this context.

The development of the male reproductive system is a continuous process that begins early in gestation and continues during the prenatal and postnatal periods, until complete structural and functional maturity at puberty [11]. Testicular development relies on a series of interconnected cell division, migration, and differentiation processes that are controlled by numerous endocrine, paracrine, and intracellular signaling pathways. As a result, testicles are susceptible to damage caused by various environmental conditions and/or endocrine disrupting chemicals that have lifelong effects on reproductive function [12]. The reproductive system exhibits a high degree of plasticity during development, when it can be “shaped” in ways that affect subsequent function. The reproductive axis may be the target of maternal over- or undernutrition, stress, and prenatal exposure to androgens, leading to altered reproductive function [1]. These programmed changes in testicular structure and function may be important in determining fertility [1].

Numerous postnatal environmental factors have a strong influence on the reproductive health of the offspring. Particular attention should be paid to pollutants, infectious agents, and unhealthy diets, which individually or in synergy can have a negative impact on male reproductive function [13,14,15]. The modern diet is characterized by a significant increase in fructose consumption, especially among the young population, which is caused by added sweeteners, sucrose, and high-fructose corn syrup. Fructose is easily absorbed, does not stimulate insulin secretion, and is rapidly metabolized in the liver, where it stimulates lipogenesis [16]. As a result, the prevalence of comorbidities continues to increase dramatically, especially in adolescents and adults. Obesity, impaired glucose homeostasis, insulin resistance, and other forms of metabolic disorders are risk factors that are highly associated with impaired reproductive function and fertility [17]. In female rats, increased fructose consumption affects the length of the estrous cycle and the histology of the ovaries and uterus [18]. Structural changes in testicular tissue; activation of inflammatory, mitotic, and apoptotic pathways; and a low sperm count are the consequences of high fructose intake in male rats [19,20,21]. Some of these disorders may be related to oxidative stress, which is caused by an imbalance between the overproduction of reactive oxygen species (ROS) and the ability of the antioxidative defense system (ADS) to eliminate free radicals [22]. Testicular tissue is highly susceptible to free-radical activity and oxidative stress due to the high rate of cell division and competition between cells for oxygen. ROS are chemically reactive molecules that are mainly formed as metabolic by-products during mitochondrial respiration and various enzymatic reactions. They have positive and negative effects on male reproduction [23]. Under controlled and balanced conditions, ROS are beneficial and promote essential physiological processes, such as sperm capacitation, acrosome reaction, and intercellular signaling [24]. However, excessive accumulation of ROS becomes pathological and triggers a range of cell damage that impairs sperm function [25]. Free radicals attack unsaturated fatty acids and cholesterol in the lipid membrane and form peroxidation products. The lipids of the cell membrane in particular are damaged. Disturbance of the homeostatic balance between ROS and the ADS can lead to the development of oxidative stress if the highly reactive molecules ROS outweigh the antioxidant defense mechanisms. Oxidative stress is a major contributor to the formation of abnormal sperm and a reduction in the sperm count, as well as alteration and fragmentation of sperm DNA, leading to infertility [26].

Prenatal glucocorticoid exposure and moderate postnatal fructose consumption as risk factors are nowadays widespread. The consequences of their combination for testicular development and function are not fully understood. We hypothesized that prenatal dexamethasone exposure in combination with moderate postnatal fructose consumption would have stronger effects on testicular structure and physiology than either factor alone. To test this hypothesis, we examined the morphometric and histologic characteristics of fetal and adult testicles and measured serum testosterone levels and antioxidant enzyme activity in the testicles of adult offspring.

## 2. Results

### 2.1. Body Weight, Testicular Weight, and Gonadosomatic Index

Exposure to dexamethasone (Dx) during the last week of fetal development (from gestational days 16 to 18) caused a significant reduction (18%; *p* < 0.0001) in body weight (bw) in 21-day-old male fetuses, while it did not affect the gestation length, litter size, male-to-female pup ratio, or pup viability (Table 1). The male offspring born spontaneously reached the body weight of the control males by adulthood. Fructose supplementation in the postnatal period did not contribute to changes in body weight in the F and DxF groups (Table 1).

The testicular weight of adult offspring prenatally exposed to Dx was not significantly altered, and fructose supplementation had no effect (Table 1).

The gonadosomatic index (GSI—calculation of the gonad weight as a proportion of the total body weight) was significantly reduced in the DxF group compared to the other experimental groups (by 17% compared to the control group (C), (*p* < 0.0001); by 18% compared to the Dx group (*p* < 0.0001); and by 14% compared to the F group (*p* < 0.01); Table 1).

### 2.2. Histomorphometric, Stereological, and Biochemical Analyses

**Figure 1:**
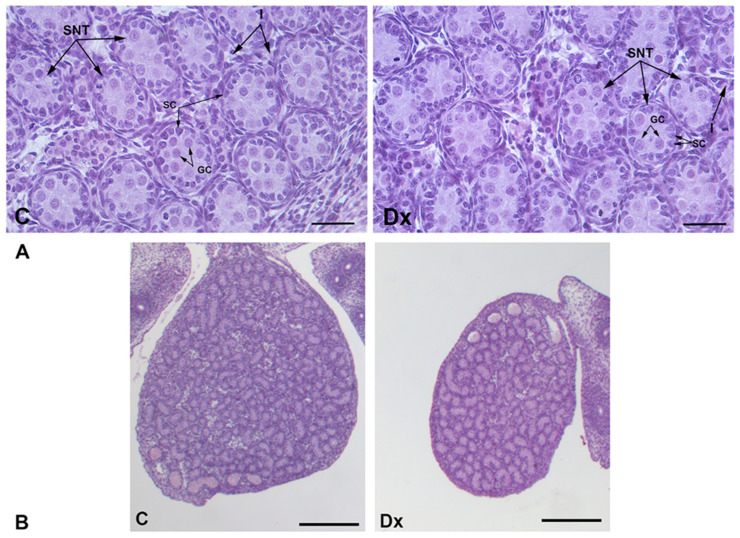
Representative micrographs of hematoxylin–eosin-stained sections of fetal testicles on day 21 of gestation in control (C) and Dx-exposed (Dx) fetuses. Testicular histology was not altered after fetal Dx exposure (**A**), but the testicular volume was significantly reduced (**B**). SNT—seminiferous tubules; I—interstitium; SC—Sertoli cells; GC—gonocytes. Scale bar (**A**) 50 µm and (**B**) 400 µm.

The fetal testicles consisted of immature seminiferous tubules with centrally located gonocytes and a peripheral rim of immature Sertoli cells, recognizable by their triangular nuclei, surrounded by a basement membrane. The interstitium contained fetal Leydig cells and undifferentiated mesenchymal cells (Figure 1A). Histological analysis of hematoxylin–eosin-stained testicular sections demonstrated that testicular histology was similar in the control group and the Dx-exposed group.

**Figure 2:**
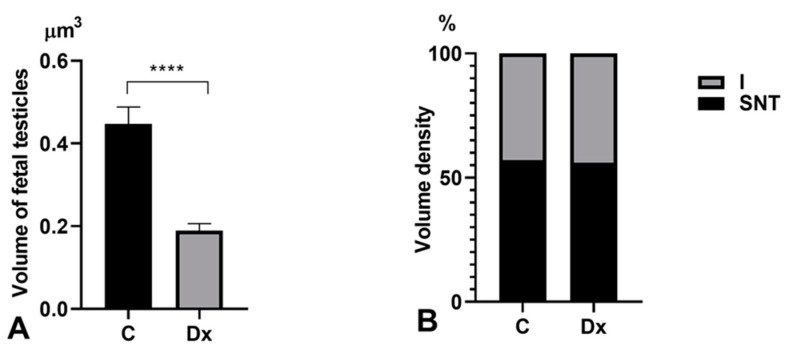
Stereological parameters: (**A**) volume of fetal testicles; (**B**) volume density (%) of seminiferous tubules (SNT) and the interstitium (I) in fetal testicles in control (C) and Dx-exposed (Dx) fetuses. Results are presented as the mean ± SD (n = 6), **** *p* < 0.0001.

Stereological examination showed that the volume of fetal testicles decreased significantly by 58% (*p* < 0.0001) after Dx treatment. The volume density of seminiferous tubules and the interstitium remained unchanged (Figure 1B and Figure 2A,B). Fetal testicular volume correlated to the body weight was significantly reduced by 44% (*p* < 0.0001) in fetuses exposed to Dx compared to control values (Table 1).

**Figure 3:**
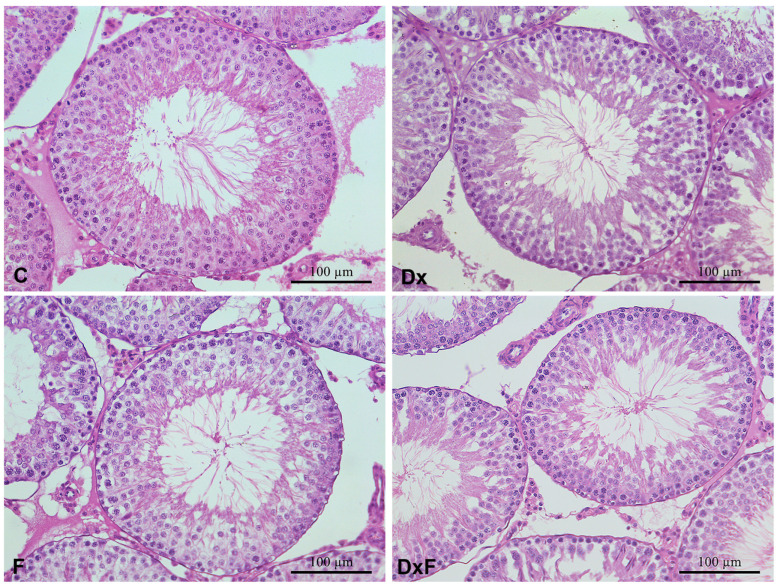
Representative micrographs of hematoxylin–eosin-stained testicular sections from adult 3-month-old offspring of C, Dx, F, and DxF groups. Testicular histology was not altered after prenatal exposure to Dx. Postnatal fructose overconsumption decreased the size of the seminiferous tubules only in offspring prenatally exposed to Dx (DxF). Scale bar 100 µm.

In the adult male offspring of the control group, the testicles were formed of large, closely spaced SNTs. The SNTs were lined with a stratified germinal epithelium with layers of developing spermatocytes and Sertoli cells. Clusters of spermatozoa could be observed in the lumen of the SNTs. The tubules were separated by interstitial tissue with Leydig cells (Figure 3). Prenatal exposure to Dx had no effect on the histological structure of the testicles of adult offspring.

**Figure 4:**
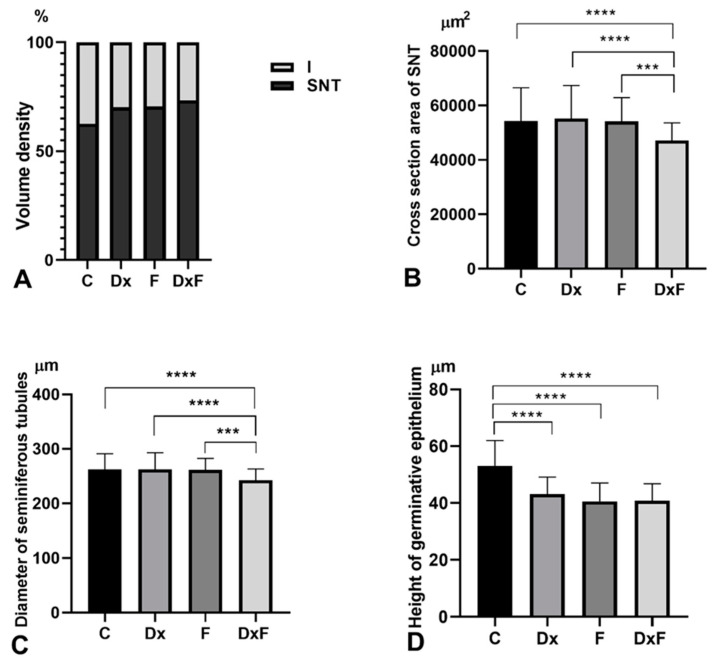
(**A**) Volume density (%); (**B**) cross-section area of SNT (µm^2^); (**C**) diameter of SNT (µm); (**D**) height of the germinative epithelium (µm) in adult testicles of C, Dx, F, and DxF groups. SNT–seminiferous tubules. Results are presented as the mean ± SD (n = 6), **** *p* < 0.0001, *** *p* < 0.001.

**Figure 5:**
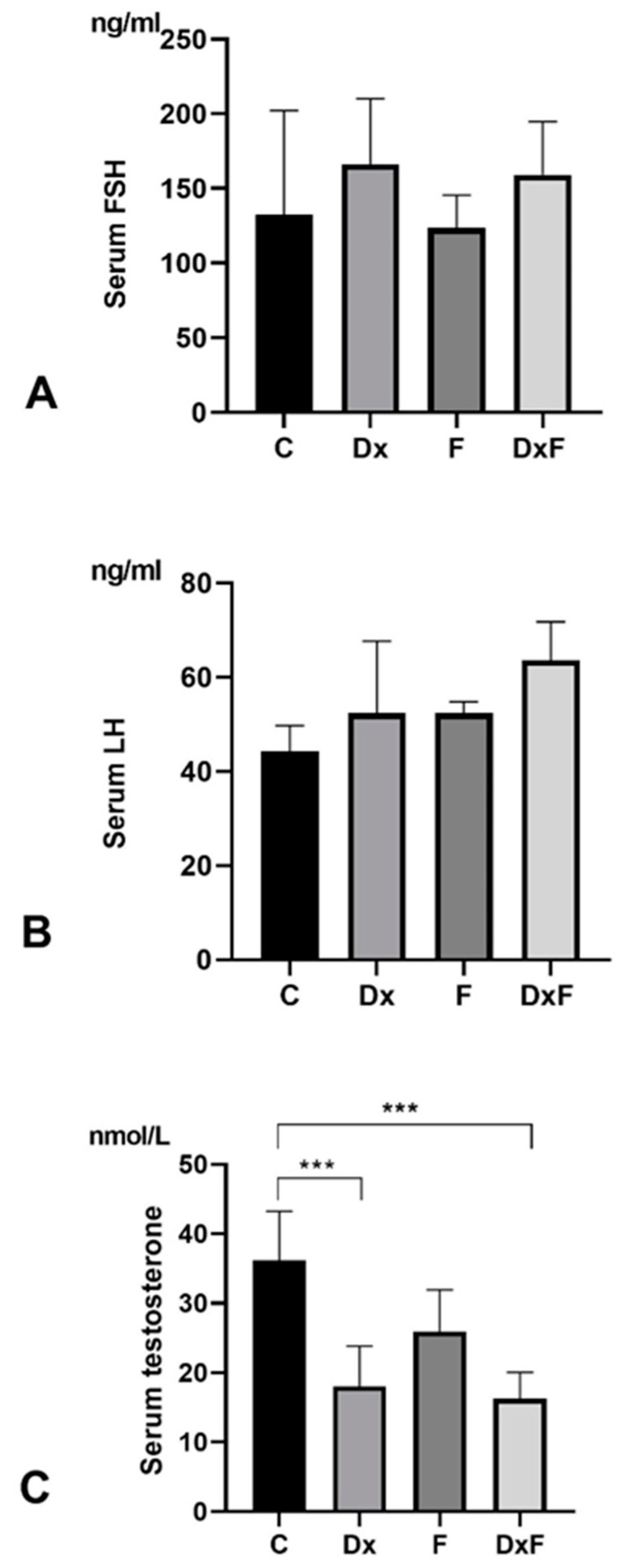
Serum concentrations of (**A**) FSH, (**B**) LH, and (**C**) testosterone in C, Dx, F, and DxF groups. Results are provided as the mean ± SD (n = 6), *** *p* < 0.001.

Stereologic and morphometric examination showed that neither prenatal exposure to Dx nor fructose supplementation in the postnatal period caused a change in the volume density of SNTs and the interstitium (Figure 4A). However, the cross-section area and diameter of the SNTs were significantly reduced in offspring prenatally overexposed to Dx and postnatally exposed to fructose overconsumption (DxF group) compared to the C group (by 15% and 8%, respectively; *p* < 0.0001), compared to the Dx group (by 16% and 8%, respectively; *p* < 0.0001), and compared to the F group (by 14% (*p* < 0.001) and 7% (*p* < 0.001), respectively; Figure 4B,C). The height of the germinative epithelium was significantly reduced in the Dx, F, and DxF groups compared to the C group by 17% (*p* < 0.0001), 22% (*p* < 0.0001), and 22% (*p* < 0.0001), respectively (Figure 4D). The serum concentrations of FSH and LH were not significantly changed (Figure 5A,B), while the serum testosterone concentrations were significantly reduced by 50% (*p* < 0.001) in the Dx group and by 55% (*p* < 0.001) in the DxF group compared to the control values (Figure 5C).

### 2.3. Oxidative Stress Parameters

The obtained results show that the activities of GSH-Px and GR were significantly lower in the Dx and F groups than in the C group, as was the activity of GR in the F group compared to that in the Dx group (*p* < 0.05). In the testicles of rats prenatally exposed to Dx and additionally challenged by fructose consumption postnatally (DxF), the activity of SOD was significantly decreased compared to that of the rats in the Dx and F groups (*p* < 0.05). Both the activity of GSH-Px and the concentration of total GSH were significantly lower in the DxF group than in the C and Dx groups (*p* < 0.05) (Table 2).

### 2.4. Discriminant Function Analysis

**Figure 6:**
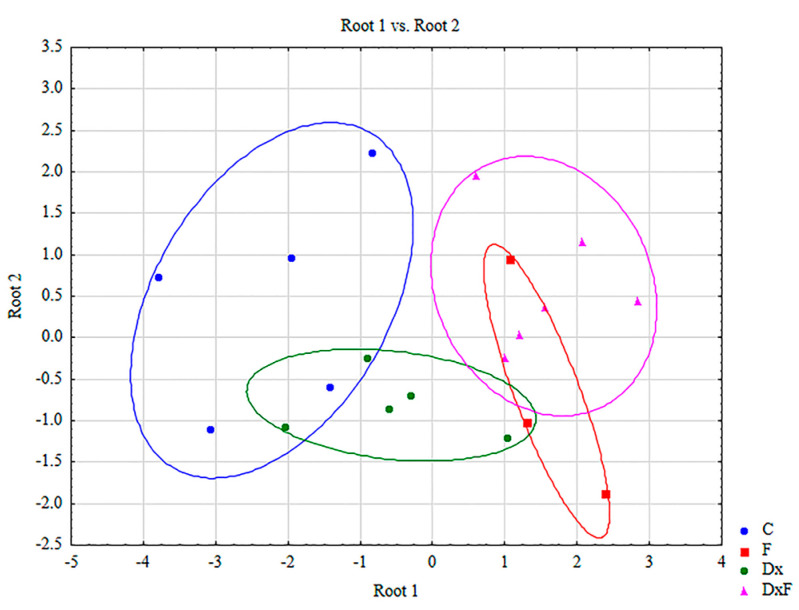
Discriminant function analysis (DA) for oxidative stress parameters in the testicles of groups C, Dx, F, and DxF. The groups were formed by root 1 (x-axis) and root 2 (y-axis). Statistical significance (the Wilks’ lambda distribution) was observed between groups.

Discriminant function analysis was used to discriminate/separate investigated groups of animals and to identify variables that contributed significantly to the differences in oxidative stress parameters between the animal groups studied. The discriminant analysis showed that there is a separation of the animal groups studied but also a partial overlap, especially between the C and Dx groups and particularly between the F and Dx groups (Figure 6). Discriminant function analysis revealed three roots. The first two roots explained 24.13% of the total variance. According to root 1 (18.74%), the control group contributed the most to the discrimination, followed by the F and DxF groups. According to root 2 (5.39%), the Dx, F, and DxF groups contributed the most to the discrimination (Table 3). The parameters contributing significantly to the discrimination of the groups were observed for GR (*p* = 0.179149), GSH (*p* = 0.256856), and SH groups (*p* = 0.480321), but in no case was statistical significance reached (Table 4). The standardized coefficients for the canonical variables are shown in Table 5.

### 2.5. Spearman Rank Order Correlations

A square matrix of Spearman rank order correlations were calculated to determine the influence of treatments on investigated parameters (Table 6).

As can be seen, the treatments correlated significantly with the activities of SOD (*p* = 0.04398), GSH-Px (*p* = 0.04266), and GR (*p* = 0.008273).

A detailed report of Spearman rank order correlations is shown in Table 7. There were positive significant correlations between SOD and GST, GR and GSH, GST and SH, and GSH and SH. The treatment itself correlated negatively with GR.

## 3. Discussion

Prenatal dexamethasone (Dx) overexposure and moderate postnatal fructose intake are common in everyday life, but the consequences of their combination for testicular structure and function have not yet been adequately studied. The results of this study showed the effects of both factors on testicular structure and redox balance in testicular tissue. The combination of prenatal Dx exposure and moderate postnatal fructose consumption has a more detrimental effect on oxidative homeostasis than either factor alone.

Normal testicular development is essential for effective fertility and reproduction in adulthood. The function of the male reproductive system depends on the structure of the gonads, a sufficient number of viable gametes, and adequate hormonal regulation. In rats, testicular development begins early, around fetal day 15.5, when many cellular and functional processes start, including the induction of somatic cell migration of the mesonephros, which forms the seminiferous tubules and Sertoli cells. Sertoli cells are required for the proper differentiation of germ cells, peritubular myoid cells, and Leydig cells. Leydig cells produce testosterone, which is necessary for the elongation of seminiferous tubules, the masculinization of the reproductive tract, and the initiation of testicular descent. The fetal developmental period is essential for the normal differentiation and masculinization of the male reproductive system [27]. Challenges from maternal environmental factors during a “window of vulnerability” could play an important role in the development of the gonads and thus in male fertility and aging reproduction.

Exposure to Dx late in gestation results in intrauterine growth retardation (IUGR), followed by reduced body weight in male fetuses on day 21 of fetal development. Fetal growth restriction is a complex and multifactorial disorder with a broad range of possible causes. Insults during prenatal life that compromise the quality of the uterine environment can impair the full genetic growth potential of the fetus and lead to IUGR [6]. The prenatal milieu with an excess of glucocorticoids disturbs the development of body weight regulatory systems, and these changes persist during birth and throughout the neonatal and prepubertal periods [28]. IUGR may be the result of the reduced oxygen and nutrient transport capacity of the placenta [29] and the altered vascular profile of the placenta and downregulated VEGF and VEGF receptor expression [6]. IUGR leads to a reduction in prenatal growth and altered ontogeny of the developing organs of the offspring, including the gonads [30,31]. If the postnatal growth path is inappropriately higher than that in utero, catch-up growth occurs [32]. Normalization of body weight in adult male offspring prenatally exposed to Dx could be the result of catch-up growth during postnatal life. The study showed that the applied metabolic challenge from weaning to adulthood, i.e., moderately increased fructose consumption mimicking unhealthy dietary habits, had no effect on the body weight of the male offspring. The fructose dosage and the time regime were not intensive enough to cause changes in body weight regulation.

The results showed that prenatal exposure to Dx during the critical period for testicular development impairs male gonadal growth, as evidenced by a reduced volume of fetal testicles, while the structure remains unchanged. Prenatally administered Dx mediates changes in the dynamic physiological balance between pro- and anti-apoptotic proteins and cell cycle proteins [33]. This could be a mechanism to control the cell number and growth rate in developing testicles [34]. The expression of key sex-determining genes for testicular development and the Leydig cell marker 3β-hydroxysteroid dehydrogenase also decreases, followed by decreased testicular and circulating testosterone levels in fetal mice [35]. These phenomena may lead to inappropriate development of the male reproductive system; alter sexual maturation, masculinization, and function in adulthood; and make the male reproductive system more susceptible to environmental changes [35]. The changes in fetal testicular volume not only reflect the overall reduced growth rate of the body but also represent a specific effect of Dx on the testicles.

Control testicular weight was reached in adult offspring exposed prenatally to dexamethasone. Decreased serum testosterone concentrations without changes in serum FSH and LH concentrations indicated a disruption of the feedback mechanism. Thus, programming by prenatal Dx exposure targets pituitary regulation and steroid synthesis of Leydig cells in the offspring [36].

Additional challenges due to fructose consumption during postnatal development led to changes in morphometric parameters. A reduced diameter of SNTs, which is, indeed, related to the height of the germinative epithelium, is a good indicator of reduced spermatogenic activity, as a direct positive correlation was found between the diameter of the SNTs and spermatogenic activity [37]. The reduction in the height of the germinative epithelium indicated that Dx and fructose alone or in combination affect the number of germ cells in seminiferous tubules and the efficiency of the spermatogenesis process. The results obtained confirm that spermatogenesis is directly affected by the deterioration of the testicular structure.

Disturbances in the activity of antioxidant enzymes could also contribute to altered male reproductive function. Prenatal Dx exposure and moderate postnatal fructose consumption decreased the activity of glutathione peroxidase (GSH-Px) and glutathione reductase (GR). Both risk factors have already been found to alter the redox status of testicular cells [38,39,40]. Reduced activity of the antioxidant defense parameters could be a programming consequence of prenatal glucocorticoid overexposure. Oxidative stress and epigenetic mechanisms are recognized as important programming mechanisms [38]. As a result, long-lasting changes in testicular physiology occur, making them more sensitive to environmental influences and oxidative stress.

Epidemiological and experimental studies indicate that high fructose consumption contributes to reduced male fertility due to increased oxidative stress [39,40]. Oxidative stress plays an important role as a mediator of damage caused by fructose metabolism. It has also been shown that increased degradation of fructose leads to a decrease in the activity of antioxidant enzymes and that oxidative stress is the most important mechanism to explain the deleterious effects of fructose [41,42]. This could indicate that the harmful effects of a high-fructose diet on the testicles are independent of weight gain.

The activity of the antioxidative defense system (ADS) was most impaired in offspring prenatally exposed to Dx accompanied by moderate postnatal fructose consumption. This was reflected in the decreased activity of key enzymes, such as superoxide dismutase (SOD), glutathione peroxidase (GSH-Px), and glutathione reductase (GR), and decreased glutathione (GSH) levels. As a result, the cell’s ability to protect itself against oxidative damage and free-radical damage reduced. In tissues such as the testicles, which are characterized by high metabolic activity and rapid cell replication, oxidative stress can be particularly damaging, highlighting the critical importance of the tissue’s antioxidant capacity [43]. Combined with structural and hormonal changes, these physiological changes can significantly impair testicular function.

In our study, the programming effects of Dx on testicular development and function are well documented. In addition, the absence of obesity following fructose consumption suggests a more direct influence of fructose or its metabolic pathways on testicular health and function. The synergistic effect of these two factors has the greatest impact on testicular structure, function, and redox balance. The controlled use of glucocorticoids during fetal development, together with the adoption of healthy dietary habits and the development of more effective antioxidant therapies, represents a promising approach to the preservation of male fertility. Further research in this area is needed as the results obtained in animals should be interpreted with caution when extrapolating to humans.

## 4. Materials and Methods

### 4.1. Animals and Experimental Design

Adult female Wistar rats (2.5 months old, n = 24) with a regular 4-day estrus cycle, kept in the facilities of the Institute for Biological Research “Siniša Stanković”—National Institute of the Republic of Serbia, Belgrade, Serbia, under standard conditions (12 h/12 h light/dark cycle, 23 ± 2 °C, 60–70% relative humidity, food and water ad libitum) were included in the experimental procedure. All animal experiments were performed in accordance with Directive 2010/63/EU on the protection of animals used for experimental and other scientific purposes and were approved by the Ethics Committee for the Use of Laboratory Animals of the Institute for Biological Research “Siniša Stanković”—National Institute of the Republic of Serbia, University of Belgrade (no. 01–1321). The experiments were conducted in accordance with Animal Research: Reporting in Vivo Experiments (ARRIVE) guidelines and guidelines on the principles of regulatory acceptance of replacement, reduction, and refinement (3R) testing approaches.

The day on which a sperm-positive vaginal smear was detected after the females had been mated overnight with fertile males was referred to as day 0 of gestation. Pregnant females (n = 24) were randomly divided into a control and an experimental group of 12 animals each. The experimental animals received a subcutaneous injection of dexamethasone (Dx) at a dose of 0.5 mg/kg/day on gestation days 16, 17, and 18, while the control animals were treated with the same amount of saline. This dosing regimen has been used previously and corresponds to the clinical exposure in humans [44,45].

To study the effects of Dx on fetal testicles, a cesarean section was performed on day 21 of gestation. The sex of the fetuses was determined by external examination (anogenital distance), and male fetuses were selected from control (n = 6) and Dx-treated (n = 6) dams. In addition, two groups were formed, control and Dx fetal groups (C and Dx), each with six male fetuses.

To examine adult testicles, the remaining pregnant females (control group, n = 6; those treated with Dx, n = 6) were delivered spontaneously on day 22 of gestation. Immediately after weaning, on postnatal day 21, male offspring were randomly selected from each of the six litters of the control group and the six litters of the Dx-treated dams. Randomization was used to avoid possible litter bias. Four groups were formed, as shown in Table 8.

The animals in the control (C) and dexamethasone (Dx) groups were fed standard commercial feed; drinking water was available ad libitum.

**Figure 7:**
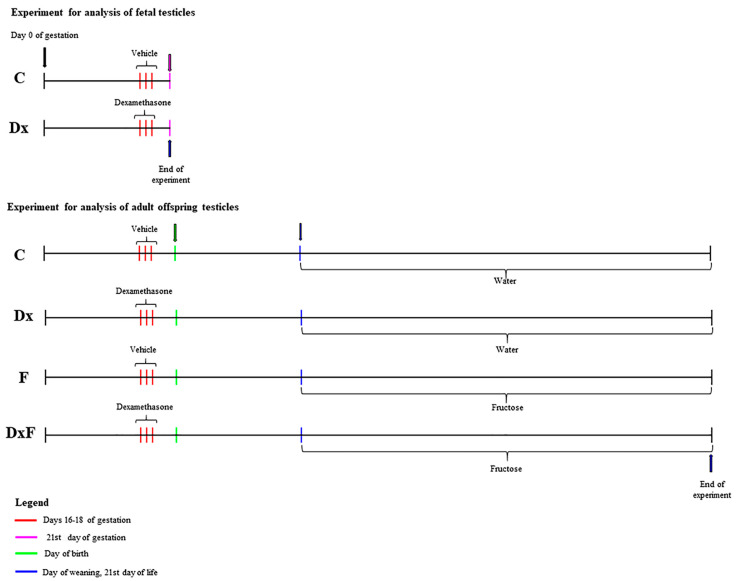
Experimental timeline.

The animals in the fructose (F) and dexamethasone+fructose (DxF) groups had ad libitum access to the same feed and a 10% (*w/v*) fructose solution instead of drinking water until the third month of life (Figure 7). The metabolic challenge applied was a moderately increased fructose consumption that mimicked unhealthy dietary habits.

All males were sacrificed in adulthood, i.e., at 3 months of age, by rapid decapitation. After decapitation, blood was collected from the body and processed for biochemical analysis. Testicular samples from fetal and adult male offspring were fixed in Bouin’s solution for 48 h, dehydrated in a series of increasing concentrations of ethanol (30%–100%), cleared in xylene, and embedded in Histowax (Histolab Product, Södra Långebergsgatan, Sweden). After the samples were embedded, each tissue block was sectioned using a rotary microtome (RM 2125RT; Leica Microsystems, Wetzlar, Germany). Five-micrometer-thick sections were prepared for quantitative stereological and histomorphometric analysis.

### 4.2. Stereological and Morphometric Analyses

**Figure 8:**
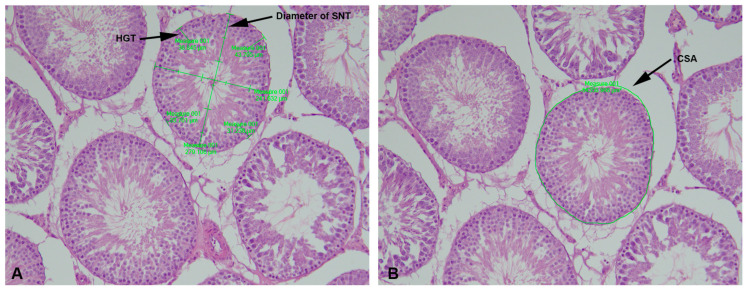
Morphometric parameters: diameter of a seminiferous tubules (SNT), height of the germinative epithelium (HE), and cross-section area (CSA); objective magnification 20×.

The volume of fetal testicles, the volume of seminiferous tubules (SNT) and the interstitium (I) of fetal and adult testicles, and their volume densities were estimated with Cavalierie´s principle [46,47] using the newCAST stereological software package Visiopharm Integrator System (VIS) version 3.2.7.0 (Visiopharm; Hørsholm, Denmark). The diameter of SNTs and the height of the germinative epithelium were measured using the distance and polygon area tools, while the cross-section areas was determined with the polygon area tool (Figure 8). Every 10th section from each of the tissue blocks was analyzed stereologically and morphometrically using a 20× objective. The first section to be analyzed was randomly selected using the random number table. These analyses were performed on 50 seminiferous tubules per animal [11,48].

### 4.3. Biochemical Analyses

The blood collected was centrifuged at 3000× *g* for 10 min, and serum samples were stored at −80 °C. Serum concentrations of gonadotropins, follicle-stimulating hormone (FSH), and luteinizing hormone (LH) were determined using enzyme-linked immunosorbent assay (ELISA) kits (Elabscience, Houston, TX, USA) for rats with catalog numbers E-EL-R0391 and E-EL-R0026 for FSH and LH, respectively. Serum levels of testosterone were determined using the chemiluminescent magnetic microparticle immunoassay (CMIA) method (Abbott, Lake County, IL, USA). The manufacturer’s instructions were strictly followed for all measurements.

### 4.4. Determination of Oxidative Stress Biomarkers in the Testicles

The testicles were dissected on ice, dried, weighed, and frozen in liquid nitrogen (−196 °C) immediately after collection, and stored at −80 °C until analysis. The tissues were minced and mixed into 5 volumes of 25 mmol/L sucrose with 10 mmol/L of Tris-HCl (pH 7.5) at 4 °C using an IKA-Werk Ultra-Turrax homogenizer (Janke and Kunkel, Staufen, Germany). This solution was mixed with 1× phosphatase inhibitor mix I and 1× protease inhibitor mix G. Homogenization was performed at 1500 rpm using the IKA-Werk Ultra-Turrax homogenizer from Janke and Kunkel (Staufen, Germany) at 4 °C. The homogenates were sonicated on ice at 10 kHz for 30 s with Bandeline Sonopuls HD 2070, followed by centrifugation in a Beckman ultracentrifuge at 100,000× *g* for 90 min at 4 °C [49]. The resulting supernatants were used to determine oxidative stress parameters. The protein concentration in the supernatants was determined according to the method of Lowry et al. [50] using bovine serum albumin as a standard and expressed as mg/g wet mass. The activity of SOD was determined by the epinephrine method [51]. One unit of SOD activity was defined as the amount of protein that causes 50% inhibition of epinephrine autoxidation at 26 °C and was expressed as specific activity (U/U/g wet mass). CAT activity was assessed by the rate of decomposition of H_2_O_2_ [52], and a unit of CAT activity was expressed as µmol H_2_O_2_/min/U/g wet mass. The activity of GSH-Px was determined after oxidation of nicotinamide adenine dinucleotide phosphate (NADPH) as the substrate with t-butyl hydroperoxide [53], and one unit of GSH-Px activity was expressed as nmol NADPH/min/U/g wet mass. The activity of GR was measured according to the method of Glatzle et al. [54]. This method is based on the ability of GR to catalyze the reduction of oxidized glutathione (GSSG) to reduced glutathione (GSH) using NADPH as a substrate in a phosphate buffer (pH 7.4). One unit of activity GR was expressed as nmol NADPH/min/U/g wet mass. The activity of GST toward 1-chloro-2,4-dinitrobenzene (CDNB) was determined according to the method of Habig et al. [55], and one unit of GST activity was expressed as nmol GSH/min/U/g wet mass. The method is based on the reaction of CDNB with the SH group of GSH catalyzed by GST in the samples.

The activity of antioxidant defense enzymes was measured in triplicate for each sample using a Shimadzu UV-1900i spectrophotometer (Shimadzu Corporation, Nishinokyo-Kuwabaracho, Nakagyo-ku, Kyoto, Japan) with a temperature-controlled cell holder. All chemicals were purchased from Sigma-Aldrich (St. Louis, MO, USA).

### 4.5. Determination of Nonenzymatic Antioxidants

The concentration of total GSH was determined at 412 nm as GSH oxidation by 5,5′-dithiobis-(2-nitrobenzoic acid) (DTNB) and NADPH reduction in the presence of GR [56] and expressed as nmol/g tissue. The concentration of sulfhydryl (SH) groups was determined with 5,5′-dithio-bis-(2-nitrobenzoic acid) (DTNB) according to the method of Ellman [57] and expressed as μmol/g wet mass. All chemicals were purchased from Sigma-Aldrich (St. Louis, MO, USA).

### 4.6. Statistical Analysis

Data were presented as the mean ± standard deviation (SD). The distribution of the data was checked using the Shapiro–Wilk test (n < 50). Identification of outliers from nonlinear regression was performed with Graph Pad Prism software (v. 5.0, USA) using the ROUT method based on the false discovery rate (FDR), with Q (maximum desired FDR) = 10% [58]. The difference between the studied groups was tested using one-way ANOVA. Because interactive effects were observed, for pairwise comparisons of multiple treatment groups, Fisher’s least significant difference (LSD) test was used, with *p* < 0.05 as the significance level, using STATISTICA software (v. 12.5, USA). The unpaired Student’s t-test was used to evaluate the differences between C and Dx fetal groups for stereological and morphometric analysis. A confidence level of *p* < 0.05, *p* < 0.01, *p* < 0.001, and *p* < 0.0001 was considered statistically significant. Figures were created using GraphPad Prism 8. Discriminant analysis (DA) was used to separate the groups under study and determine the variables that contributed most to separating the groups. DA is used to analyze the data between the dependent variable, which is categorical (parameters studied), and the predictor or independent variable, which is an interval (different treatments) [59]. Spearman rank order correlations were also performed. For all tests, *p* < 0.05 was considered statistically significant.

## 5. Conclusions

A comparison of the effects of both treatments and their potential synergistic effects suggests that prenatal exposure to dexamethasone acts as a strong programming factor influencing the developmental trajectory of testicles from the fetus to adulthood. This confirmed Dx-related changes in morphometric parameters, decreased serum testosterone levels, and reduced antioxidant defense system activity in adult offspring prenatally exposed to dexamethasone. Moderate postnatal fructose intake also alters morphometric parameters and oxidative homeostasis in adult testicles. However, the synergistic effect of these two factors causes more deleterious changes in testicular tissue. Impaired redox homeostasis and structural changes in adult testicles and reduced testosterone levels indicate reduced testicular function in adult offspring. The continuation of this study will show whether the changes caused by fructose are reversible, whether these treatments may have transgenerational effects, and whether there is a sex-specific response to these treatments.

## Figures and Tables

**Table 1:** Gestational length, litter size, female–male ratio, fetal and adult body weights, testicular weight, gonadosomatic index, and volume of fetal testicles expressed per body weight unit in the control group (C) and in the dexamethasone (Dx), fructose (F), and dexamethasone+fructose (DxF) groups.

	Gestational Length (days)	Litter Size	Female–Male Ratio	Fetal bw (g)	Adult bw (g)	Testicular Weight (g)	GSI (gw/bw ∗ 100)	Vft/bw
C	22 ± 0	11 ± 2	1:1 ± 0.1	5.5 ± 0.4	322 ± 54	1.49 ± 0.13	0.46 ± 0.05	0.09 ± 0.007
Dx	22 ± 0	11 ± 3	1:1 ± 0.3	4.3 ± 0.4 ^A^	314 ± 30	1.46 ± 0.12	0.46 ± 0.03	0.05 ± 0.003 ^A^
F					370 ± 63	1.63 ± 0.14	0.44 ± 0.03	
DxF					383 ± 42	1.46 ± 0.17	0.38 ± 0.03 ^B,C,D^	

All results are provided as the mean ± SD (n = 6); significant differences: ^A^ dexamethasone (Dx) vs. control (C), *p* < 0.0001; ^B^ dexamethasone+fructose (DxF) vs. control (C), *p* < 0.0001; ^C^ dexamethasone+fructose (DxF) vs. dexamethasone (Dx), *p* < 0.0001; ^D^ dexamethasone+fructose (DxF) vs. fructose (F), *p* < 0.01.

**Table 2:** The activities of superoxide dismutase (SOD), catalase (CAT), glutathione peroxidase (GSH-Px), glutathione reductase (GR), and glutathione S-transferase (GST), as well as total glutathione (GSH) and sulfhydryl group (SH) concentrations in the adult testicles of groups C, Dx, F, and DxF.

	C	Dx	F	DxF
SOD (U/g wet mass)	12,578.9 ± 857.9	13,076.53 ± 1834.35	15,474.56 ± 13,076.64	9744.31 ± 3282.78 ^E,F^
CAT (μmol H_2_O_2_/min/g wet mass)	857.9 ± 145.29	754.47 ± 52.37	1158.03 ± 708.59	911.01 ± 420.92
GSH-Px (μmol NADPH/min/g wet mass)	134,797.4 ± 34,722.1	91,157 ± 41738.28 ^A^	66,945.32 ± 23,556.9 ^B^	78,472.65 ± 24,074.25 ^C^
GR (nmol NADPH/min/g wet mass)	1783.8 ± 1090.36	1186.49 ± 630.84 ^A^	428.30 ± 132.61 ^B,D^	325.08 ± 174.99 ^C,E^
GST (nmol GSH/min/g wet mass)	90,630.0 ± 15,689.36	99,731.25 ± 6062.29	95,837.50 ± 27,172.89	88,337.50 ± 9785.61
GSH (µmol/g wet mass)	3253.6 ± 1237.35	3319.92 ± 499.33	2536.06 ± 317.59	2123.17 ± 480.77 ^C,E^
SH (nmol/g wet mass)	719.6 ± 120.83	828.42 ± 118.18	883.60 ± 310.24	707.92 ± 104.80

The data are expressed as the mean ± SD. The one-way ANOVA post-hoc Fisher’s least significant difference (LSD) test for pairwise comparisons of several treatment groups with *p* < 0.05 as the level of significance. Significant differences between groups: ^A^ Dx vs. C; ^B^ F vs. C; ^C^ DxF vs. C; ^D^ Dx vs. F; ^E^ Dx vs. DxF; ^F^ DxF vs. F.

**Table 3:** Mean values of canonical variables: contribution of the analyzed groups to the discrimination.

Group	Root 1	Root 2
C	−2.22189	0.451185
F	1.57741	−0.644622
Dx	−0.57059	−0.811620
DxF	1.53837	0.622674

Root 1: C > F > DxF > Dx. Root 2: Dx > F > DxF > C.

**Table 4:** Parameters that contributed to the discrimination between the groups studied.

Variable	Wilks’ Lambda	Partial Lambda	F-Remove (3.9)	*p*-Value	Toler.	1-Toler. (R-Sqr.)
SOD	0.146698	0.878118	0.416399	0.745528	0.622707	0.377293
CAT	0.140828	0.914720	0.279694	0.838790	0.771553	0.228447
GSH-Px	0.164153	0.784747	0.822889	0.513479	0.792215	0.207785
GR	0.216311	0.595523	2.037586	0.179149	0.868542	0.131458
GST	0.134973	0.954403	0.143325	0.931384	0.538255	0.461745
GSH	0.197505	0.652228	1.599618	0.256856	0.568103	0.431897
SH	0.167259	0.770174	0.895226	0.480321	0.560317	0.439683

Discriminant function analysis summary: number of variables in the model: 7; grouping: 4 groups; Wilks’ Lambda: 0.12882; approx. F (21.26) = 3.3090; *p* < 0.2550.

**Table 5:** Standardized coefficients for canonical variables.

Variable	Root 1	Root 2
SOD	−0.309496	−0.143654
CAT	0.097974	0.265807
GSH-Px	−0.400054	0.651859
GR	−0.766891	−0.226217
GST	0.282339	0.097549
GSH	−0.848148	−0.429790
SH	0.536050	−0.741471
Eigenval.	3.198399	0.525628
Cum. prop.	0.812603	0.946147

Cum. prop.—cumulative proportion.

**Table 6:** Spearman rank order correlations between treatments and investigated parameters.

Pair of Variables	Valid N	Spearman R	t(N-2)	*p*-Value
Treatments and SOD	18	−0.431228	−1.91180	0.043980 *
Treatments and CAT	18	−0.148844	−0.60208	0.555556
Treatments and GSH-Px	19	−0.420989	−1.91362	0.042660 *
Treatments and GR	19	−0.586733	−2.98743	0.008273 *
Treatments and GST	18	0.003212	0.01285	0.989906
Treatments and GSH	19	−0.403713	−1.81941	0.086506
Treatments and SH	19	−0.057284	−0.23657	0.815814

* The parameters that contributed the most are marked with an asterisk, and are significant at *p* < 0.05.

**Table 7:** Spearman rank order correlations between investigated parameters.

	SOD	CAT	GSH-Px	GR	GST	GSH	SH
	−0.431228	−0.148844	−0.420989	−0.586733 *	0.003212	−0.403713	−0.057284
SOD		0.080882	0.010325	0.416322	0.484366 *	0.408880	0.415075
CAT	0.080882		0.029928	−0.214765	0.036765	−0.122807	−0.021672
GSH-Px	0.010325	0.029928		0.314173	−0.106295	0.428070	0.192982
GR	0.416322	−0.214765	0.314173		0.162106	0.475647 *	0.103554
GST	0.484366 *	0.036765	−0.106295	0.162106		0.364293	0.525284 *
GSH	0.408880	−0.122807	0.428070	0.475647 *	0.364293		0.522807 *
SH	0.415075	−0.021672	0.192982	0.103554	0.525284 *	0.522807 *	

* The parameters that contributed the most are marked with an asterisk, and are significant at *p* < 0.05.

**Table 8:** Schematic representation of experimental groups.

	Offspring from Control Dams	Offspring from Dx-Treated Dams
Standard commercial feed/drinking water ad libitum	C (n = 6)	Dx (n = 6)
Standard commercial feed/10% (*w/v*) fructose solution	F (n = 6)	DxF (n = 6)

## Data Availability

The data supporting the conclusions of this article will be made available by the authors upon reasonable request.

## References

[1] Evans N.P., Bellingham M., Robinson J.E. (2016). Prenatal programming of neuroendocrine reproductive function. Theriogenology.

[2] Rabadán-Diehl C., Nathanielsz P.J. (2013). From Mice to Men: Research models of developmental programming. Dev. Orig. Health Dis..

[3] Hacking D., Watkins A., Fraser S., Wolfe R., Nolan T. (2001). Respiratory distress syndrome and birth order in premature twins. Arch. Dis. Child. Fetal Neonatal. Ed..

[4] Benediktsson R., Lindsay R.S., Noble J., Seckl J.R., Edwards C.R.V. (1993). Glucocorticoid exposure in utero: New model for adult hypertension. Lancet.

[5] Green B.B., Armstrong D.A., Lesseur C., Paquette A.G., Guerin D.J., Kwan L.E., Marsit C.J. (2015). The Role of Placental 11-Beta Hydroxysteroid Dehydrogenase Type 1 and Type 2 Methylation on Gene Expression and Infant Birth Weight. Biol. Reprod..

[6] Arias A., Schander J.A., Bariani M.V., Correa F., Domínguez Rubio A.P., Cella M., Cymeryng C.B., Wolfson M.L., Franchi A.M., Aisemberg J. (2021). Dexamethasone-induced intrauterine growth restriction modulates expression of placental vascular growth factors and fetal and placental growth. Mol. Hum. Reprod..

[7] Dagklis T., Sen C., Tsakiridis I., Villalaín C., Allegaert K., Wellmann S., Kusuda S., Serra B., Sanchez Luna M., Huertas E. (2022). The use of antenatal corticosteroids for fetal maturation: Clinical practice guideline by the WAPM-World Association of Perinatal Medicine and the PMF-Perinatal Medicine foundation. J. Perinat. Med..

[8] Murphy V.E., Fittock R.J., Zarzycki P.K., Delahunty M.M., Smith R., Clifton V.L. (2007). Metabolism of synthetic steroids by the human placenta. Placenta.

[9] Wang J., Chen F., Zhu S., Li X., Shi W., Dai Z., Hao L., Wang X.J. (2022). Adverse effects of prenatal dexamethasone exposure on fetal development. Reprod. Immunol..

[10] COVID-19 Treatment Guidelines Panel (2024). Coronavirus Disease 2019 (COVID-19) Treatment Guidelines. National Institutes of Health. https://www.fondazionemisi.it/images/Covid19_Treatment_Guidelines_NIH.pdf.

[11] Jeje S.O., Raji Y. (2017). Maternal treatment with dexamethasone during gestation alters sexual development markers in the F1 and F2 male offspring of Wistar rats. J. Dev. Orig. Health Dis..

[12] Li H., Spade D.J. (2021). Reproductive toxicology: Environmental exposures, fetal testis development and function: Phthalates and beyond. Reproduction.

[13] Medaglia D.S.A., Vieira H.R., Silveira S.D.S., Siervo G.E.M.L., Marcon M.S.D.S., Mathias P.C.F., Fernandes G.S.A. (2022). High-fructose diet during puberty alters the sperm parameters, testosterone concentration, and histopathology of testes and epididymis in adult Wistar rats. J. Dev. Orig. Health Dis..

[14] Lettieri G., Marra F., Moriello C., Prisco M., Notari T., Trifuoggi M., Giarra A., Bosco L., Montano L., Piscopo M. (2020). Molecular Alterations in Spermatozoa of a Family Case Living in the Land of Fires. A First Look at Possible Transgenerational Effects of Pollutants. Int. J. Mol. Sci..

[15] Montano L., Donato F., Bianco P.M., Lettieri G., Guglielmino A., Motta O., Bonapace I.M., Piscopo M. (2021). Air Pollution and COVID-19: A Possible Dangerous Synergy for Male Fertility. Int. J. Environ. Res. Public Health.

[16] Basciano H., Federico L., Adeli K. (2005). Fructose, insulin resistance, and metabolic dyslipidemia. Nutr. Metab..

[17] Bhat S.F., Pinney S.E., Kennedy K.M., McCourt C.R., Mundy M.A., Surette M.G., Sloboda D.M., Simmons R.A. (2021). Exposure to high fructose corn syrup during adolescence in the mouse alters hepatic metabolism and the microbiome in a sex-specific manner. J. Physiol..

[18] Ko E.A., Kim H.R., Kim Y.B., Kim H.S., Lee S.H. (2017). Effect of high fructose corn syrup (HFCS) intake on the female reproductive organs and lipid accumulation in adult rats. Dev. Reprod..

[19] Yildirim O.G., Sumlu E., Aslan E., Koca H.B., Pektas M.B., Sadi G., Akar F. (2019). High-fructose in drinking water initiates activation of inflammatory cytokines and testicular degeneration in rat. Toxicol. Mech. Methods.

[20] Yildirim O.G., Guney C., Alcigir M.E., Akar F. (2023). High-fructose consumption suppresses insulin signaling pathway accompanied by activation of macrophage and apoptotic markers in rat testis. Reprod. Biol..

[21] Akar F., Yildirim O.G., Yucel Tenekeci G., Tunc A.S., Demirel M.A., Sadi G. (2022). Dietary high-fructose reduces barrier proteins and activates mitogenic signalling in the testis of a rat model: Regulatory effects of kefir supplementation. Andrologia.

[22] Aslankoc R., Ozmen O. (2019). The effects of high-fructose corn syrup consumption on testis physiopathology-The ameliorative role of melatonin. Andrologia.

[23] Checa J., Aran J.M. (2020). Reactive Oxygen Species: Drivers of Physiological and Pathological Processes. J. Inflamm. Res..

[24] De Lamirande E., Jiang H., Zini A., Kodama H., Gagnon C. (1997). Reactive oxygen species and sperm physiology. Rev. Reprod..

[25] Bansal A.K., Bilaspuri G.S. (2010). Impacts of oxidative stress and antioxidants on semen functions. Vet. Med. Int..

[26] Asadi A., Ghahremani R., Abdolmaleki A., Rajaei F. (2021). Role of sperm apoptosis and oxidative stress in male infertility: A narrative review. Int. J. Reprod. Biomed..

[27] Zambrano E., Nathanielsz P.W., Rodríguez-González G.L. (2021). Developmental programming and ageing of male reproductive function. Eur. J. Clin. Investig..

[28] Iwasa T., Matsuzaki T., Munkhzaya M., Tungalagsuvd A., Kawami T., Murakami M., Yamasaki M., Kato T., Kuwahara A., Yasui T. (2014). Prenatal exposure to glucocorticoids affects body weight, serum leptin levels, and hypothalamic neuropeptide-Y expression in pre-pubertal female rat offspring. Int. J. Dev. Neurosci..

[29] Guo J., Fang M., Zhuang S., Qiao Y., Huang W., Gong Q., Xu D., Zhang Y., Wang H. (2020). Prenatal dexamethasone exposure exerts sex-specific effect on placental oxygen and nutrient transport ascribed to the differential expression of IGF2. Ann. Transl. Med..

[30] Ristić N., Severs W., Nestorović N., Jarić I., Manojlović-Stojanoski M., Trifunović S., Pendovski L., Milošević V. (2016). Effects of prenatal dexamethasone on the rat pituitary gland and gonadotropic cells in female offspring. Cells Tissues Organs.

[31] Pampanini V., Germani D., Puglianiello A., Stukenborg J.-B., Reda A., Savchuk I., Rós Kjartansdóttir K., Cianfarani S., Söder O. (2016). Impact of uteroplacental insufficiency on postnatal rat male gonad. J. Endocrinol..

[32] Lim K., Armitage J.A., Stefanidis A., Oldfield B.J., Black M.J. (2011). IUGR in the absence of postnatal “catch-up” growth leads to improved whole body insulin sensitivity in rat offspring. Pediatr. Res..

[33] Gruver-Yates A., Cidlowski J. (2013). Tissue-specific actions of glucocorticoids on apoptosis: A double-edged sword. Cells.

[34] Gao H.B., Tong M.H., Hu Y.Q., Guo Q.S., Ge R., Hardy M.P. (2002). Glucocorticoid induces apoptosis in rat leydig cells. Endocrinology.

[35] Yun H.J., Lee J.Y., Kim M.H. (2016). Prenatal exposure to dexamethasone disturbs sex-determining gene expression and fetal testosterone production in male embryos. Biochem. Biophys. Res. Commun..

[36] Jeje S.O., Ola-Mudathir F.K., Raji Y. (2017). Experimental maternal treatment with dexamethasone during lactation induces neonatal testicular and epididymal oxidative stress; Implications for early postnatal exposure. Pathophysiology.

[37] Ferrari F., de Paiva Foletto M., Franzói de Moraes S.M., Barnabé Peres S., Segatelli T.M., Mareze da Costa C.E. (2013). Testis morphophysiology of rats treated with nandrolone decanoate and submitted to physical training. Acta. Sci. Health Sci..

[38] Thompson L.P., Al-Hasan Y. (2012). Impact of oxidative stress in fetal programming. J. Pregnancy.

[39] Gaby A.R. (2005). Adverse effects of dietary fructose. Altern. Med. Rev..

[40] Lê K.A., Tappy L. (2006). Metabolic effects of fructose. Curr. Opin. Clin. Nutr. Metab. Care.

[41] Castro M.C., Massa M.L., Arbeláez L.G., Schinella G., Gagliardino J.J., Francini F. (2015). Fructose-induced inflammation, insulin resistance and oxidative stress: A liver pathological triad effectively disrupted by lipoic acid. Life Sci..

[42] Reddy S.S., Ramatholisamma P., Karuna R., Saralakumari D. (2009). Preventive effect of Tinospora cordifolia against high-fructose diet-induced insulin resistance and oxidative stress in male wistar rats. Food Chem. Toxico..

[43] Turner T.T., Lysiak J.J. (2008). Oxidative stress: A common factor in testicular dysfunction. 2008. J. Androl..

[44] Roberts D., Dalziel S. (2006). Antenatal corticosteroids for accelerating fetal lung maturation for women at risk of preterm birth. Cochrane Database Syst. Rev..

[45] Carbone D.L., Zuloaga D.G., Hiroi R., Foradori C.D., Legare M.E., Handa R.J. (2012). Prenatal dexamethasone exposure potentiates dietinduced hepatosteatosis and decreases plasma IGF-I in a sex-specific fashion. Endocrinology.

[46] Gundersen H.J., Jensen E.B. (1987). The efficiency of systematic sampling in stereology and its prediction. J. Microsc..

[47] Ristić N., Nestorović N., Manojlović-Stojanoski M., Filipović B., Šošić-Jurjević B., Milošević V., Sekulić M. (2008). Maternal dexamethasone treatment reduces ovarian follicle number in neonatal rat offspring. J. Microsc..

[48] Pallarés M.E., Adrover E., Baier C.J., Bourguignon N.S., Monteleone M.C., Brocco M.A., González-Calvar S.I., Antonelli M.C. (2013). Prenatal maternal restraint stress exposure alters the reproductive hormone profile and testis development of the rat male offspring. Stress.

[49] Abiaka C., Al-Awadi F., Olusi S. (2000). Effect of Prolonged Storage on the Activities of Superoxide Dismutase, Glutathione Reductase, and Glutathione Peroxidase. Clin. Chem..

[50] Lowry O.H., Rosebrough N.L., Farr A.L., Randall R.I. (1951). Protein measurement with Folin phenol reagent. J. Biol. Chem..

[51] Misra H.P., Fridovich I. (1972). The role of superoxide anion in the autoxidation of epinephrine and simple assay for superoxide dismutase. J. Biol. Chem..

[52] Claiborne A., Greenwald R.A. (1985). Catalase activity. CRC Handbook of Methods for Oxygen Radical Research.

[53] Tamura M., Oschino N., Chance B. (1982). Some characteristics of hydrogen and alkyl-hydroperoxides metabolizing systems in cardiac tissue. J. Biochem..

[54] Glatzle D., Vulliemuier J.P., Weber F., Decker K. (1974). Glutathione reductase test with whole blood a convenient procedure for the assessment of the riboflavin status in humans. Experientia.

[55] Habig W.H., Pubst M.J., Jakoby W.B. (1974). Glutathione S-transferase. J. Biol. Chem..

[56] Griffith O.W. (1980). Determination of glutathione and glutathione disulfide using glutathione reductase and 2-vinylpyridine. Anal. Biochem..

[57] Ellman G.L. (1959). Tissue sulfhydryl groups. Arch. Biochem. Biophys..

[58] Motulsky H.M., Brown R.E. (2006). Detecting outliers when fitting data with nonlinear regression–a new method based on robust nonlinear regression and the false discovery rate. BMC Bioinform..

[59] Darlington R.B., Weinsberg S., Walberg H. (1973). Canonical variate analysis and related techniques. Rev. Edu. Res..

